# The location of Australian Buruli ulcer lesions—Implications for unravelling disease transmission

**DOI:** 10.1371/journal.pntd.0005800

**Published:** 2017-08-18

**Authors:** Arvind Yerramilli, Ee Laine Tay, Andrew J. Stewardson, Peter G. Kelley, Emma Bishop, Grant A. Jenkin, Mike Starr, Janine Trevillyan, Andrew Hughes, N Deborah Friedman, Daniel P. O’Brien, Paul D. R. Johnson

**Affiliations:** 1 University of Melbourne, Department of Medicine, Austin Health, Victoria, Australia; 2 Department of Medicine and Infectious Diseases, Royal Melbourne Hospital, University of Melbourne, Parkville, Victoria, Australia; 3 Health Protection Branch, Department of Health & Human Services, Melbourne, Victoria, Australia; 4 Infectious Diseases Department, Austin Health, Heidelberg, Victoria, Australia; 5 Department of Infectious Diseases, Peninsula Health, Frankston, Victoria, Australia; 6 Department of Infectious Diseases, Monash Health, Clayton, Victoria, Australia; 7 Department of General Medicine, The Royal Children's Hospital, Parkville, Victoria, Australia; 8 Department of Infectious Diseases, Alfred Health, Prahran, Victoria, Australia; 9 Department of Infectious Diseases, Barwon Health, Geelong, Victoria, Australia; Swiss Tropical and Public Health Institute, SWITZERLAND

## Abstract

**Background:**

Buruli ulcer (BU), caused by M*ycobacterium ulcerans*, is increasing in incidence in Victoria, Australia. To improve understanding of disease transmission, we aimed to map the location of BU lesions on the human body.

**Methods:**

Using notification data and clinical records review, we conducted a retrospective observational study of patients diagnosed with BU in Victoria from 1998–2015. We created electronic density maps of lesion locations using spatial analysis software and compared lesion distribution by age, gender, presence of multiple lesions and month of infection.

**Findings:**

We examined 579 patients with 649 lesions; 32 (5.5%) patients had multiple lesions. Lesions were predominantly located on lower (70.0%) and upper (27.1%) limbs, and showed a non-random distribution with strong predilection for the ankles, elbows and calves. When stratified by gender, upper limb lesions were more common (OR 1·97, 95% CI 1·38–2·82, p<0·001) while lower limb lesions were less common in men than in women (OR 0·48, 95% CI 0·34–0·68, p<0·001). Patients aged ≥ 65 years (OR 3·13, 95% CI 1·52–6·43, p = 0·001) and those with a lesion on the ankle (OR 2·49, 95% CI 1·14–5·43, p = 0·02) were more likely to have multiple lesions. Most infections (71.3%) were likely acquired in the warmer 6 months of the year.

**Interpretation:**

Comparison with published work in Cameroon, Africa, showed similar lesion distribution and suggests the mode of *M*. *ulcerans* transmission may be the same across the globe. Our findings also aid clinical diagnosis and provide quantitative background information for further research investigating disease transmission.

## Author summary

Buruli ulcer is an emerging tropical disease that is also increasingly common in the temperate Australian state of Victoria. The mode of transmission of this geographically restricted infection remains elusive. We have accurately mapped the location of 649 PCR-confirmed Buruli lesions affecting 579 patients and displayed their position on front and back human body diagrams. Lesion distribution density was assessed with computer-generated heat-maps. Buruli lesion distribution was most common on exposed parts of the body (distal limbs). However, even on exposed areas, lesion distribution was highly unevenly distributed and focused towards ankles, backs of calves and elbows. The palmar and plantar surfaces of hands and feet were rarely affected. We propose that targeting behavior by biting insects rather than direct contact with a contaminated environment best explains the lesion distribution we observed.

## Introduction

Buruli ulcer (BU), listed by the World Health Organisation (WHO) as a neglected tropical disease, is a destructive infection of the subcutaneous tissue caused by the acid-fast bacillus *Mycobacterium ulcerans* [1,2]. It is endemic in at least 33 countries and is the third most prevalent mycobacterial disease worldwide after tuberculosis and leprosy [3]. The natural history of BU begins as a small nodule or plaque that usually progresses into a large, necrotic ulcer if left untreated. In severe cases, the disease can result in significant cosmetic and functional deformities. Despite vastly different climatic and socioeconomic conditions, West Africa and the Australian state of Victoria are the two most commonly affected regions globally [4]. In Victoria BU occurs in well-defined endemic zones including the Mornington and Bellarine Peninsulas near Melbourne [5]. These are coastal regions in a climactically temperate region where the annual incidence of BU has progressively increased since the mid-1990s [5–8].

A characteristic of BU is its sharp geographical restriction, often to quite small areas of just a few square kilometres [5]. However, the reservoir and mechanisms of disease transmission are yet to be determined. There are two general theories of transmission. The first is inoculation from a contaminated environmental source through contact with sharp leaves, thorns and bushes (for example), or through direct exposure of existing wounds [4]. The second is inoculation via an insect vector which has itself been previously contaminated [4]. As the pathogen grows best at temperatures slightly lower than core body temperature, some researchers have also considered a third possibility involving acquisition of *M*. *ulcerans* by aerosol or inoculation, followed by silent dissemination and local reactivation at relatively cooler peripheral body sites [9,10].

The distribution of BU lesions on the human body was investigated recently in Cameroon using spatial analysis in 88 confirmed cases [11]. The main finding was a non-random distribution with lesions tending to cluster at large joints such as the ankles and elbows. These observations support previous research indicating the upper and lower limbs are the most commonly affected body sites [6,12]. Using a similar method, we have conducted our own investigation on a much larger cohort of confirmed cases from Victoria. We aimed to quantitatively assess lesion location to better understand transmission and to define differences and similarities between disease in temperate and tropical zones. We also intended to determine more comprehensively the clinical pattern of disease presentation in Australian populations to assist clinicians recognise BU earlier and minimise morbidity.

## Methods

### Study design & definitions

This was a multisite, retrospective, observational study of patients diagnosed with BU in Victoria. We aimed to include all cases from 1 January 2004 when the disease was first made legally notifiable by the Department of Health & Human Services (DHHS) until December 31, 2015. Notifications prior to January 2004 were voluntary. DHHS keeps records of cases including clinical and epidemiological information as part of their enhanced surveillance program. Additional cases diagnosed between 1998 and 2003 by clinicians involved in the study were also included. Cases were confirmed either by a positive culture or *IS2404* PCR for *M*. *ulcerans* (generally both) [13]. If patients developed additional BU lesions more than 12 months apart, these were defined as re-infections and included as separate BU episodes.

### Data collection & mapping

Primary data collected for all patients included the physical location of lesions on the body, gender, patient age at diagnosis, and the geographic endemic region where the patient was most likely infected. Endemic areas comprised the Bellarine Peninsula, the Mornington Peninsula, and ‘Other’ made up of Phillip Island, the Frankston Area, Geelong, East Gippsland, Melbourne’s South East Suburbs and Interstate endemic regions. When patients had travelled or resided in multiple different endemic areas they were excluded from the sub-analyses that compared patients by region.

To increase the accuracy of lesion location descriptions, we contacted clinicians responsible for treating patients from seven major tertiary hospitals in Victoria, and requested they map the location of lesions from their own records in a systematic, standardised manner. Clinicians used a variety of sources to identify lesion locations including clinical notes, referral letters, pathology reports and photographic evidence. When lesions were large, we asked them to locate the likely initial origin and map this point. All mapping from clinicians was initially completed on hard-copy ‘front’ and ‘back’ templates of the human body which were printed onto standard A4 graph paper.

Coordinates for each lesion were then collected and recorded electronically. Front and back shapefiles were created separately by inputting x and y coordinates from outlines of the hardcopy templates and adding this data to blank ArcGIS maps. Coordinates of lesions were then inputted and similarly added as another layer to the previously created templates. A kernel density analysis and raster clipping tool within the software was used to visualise lesion distribution. All electronic mapping and spatial analysis was conducted using ESRI’s ArcGIS ArcMap (Economic and Social Research Institute, Redlands, USA, version 10·3·1), RStudio (RStudio, Boston, USA, version 0·99·893) and R (The R Foundation for Statistical Computing, version 3·3·1).

### Statistical analysis

The localisation of lesions was categorised into single specific body regions (e.g. foot, ankle) or groups of specific body regions. These were then analysed to determine if there were specific distribution patterns that varied by gender, age, the presence of multiple lesions or by average maximal daily temperatures for the endemic area. The groups of specific body regions included; upper limb (hand, wrist, forearm, elbow, arm, shoulder); lower limb (foot, ankle, leg, knee, thigh, buttocks); distal lesions (hand, wrist, forearm, elbow, foot, ankle, leg, knee); proximal lesions (arm, shoulder, thigh, buttock, face, neck, abdomen, back, chest); arm and shoulder combined; hand, wrist, forearm and elbow combined.

To determine the likely date of infection we analysed a subset of patients from one health service (Barwon Health) with known duration of symptoms prior to presentation. The estimated date of infection was determined by subtracting from the date of diagnosis the number of days of symptoms prior to diagnosis and the estimated mean incubation time for *M*. *ulcerans* in Victoria (135 days) [5]. We then categorised the dates of estimated infection in groups of 3 months from hottest to coldest according to the average daily temperatures in Victoria from the Australian Bureau of Meteorology [14]. Associations between the hottest 3 months and the coldest 6 months were determined using univariate analyses with the two coldest month categories combined (May to October) to provide a sample size large enough to allow meaningful comparative analyses.

Data was analysed using STATA 14 (StataCorp, Texas, USA). Univariate analyses were performed using Mantel-Haenszel and multivariate analyses were performed using logistical regression analyses. A p-value of less than 0.05 was deemed significant.

### Ethical statement

Our study was performed as part of the ongoing enhanced surveillance program conducted by the Department of Health and Human Services of Victoria (DHHS). Low risk ethics approvals were obtained from the Institutional Review Board at each participating clinical site to allow us to access any missing demographic patient data and permit the most accurate possible lesion location through each patient’s treating clinician (IRBs: Austin Health, Barwon Health, Peninsula Health, Monash Health, Royal Children’s Hospital, Melbourne Health, Alfred Health). Institutional Review Boards did not require us to obtain consent as data we were collating and analysing had already been notified to DHSS under the *Public Health and Wellbeing Act 2008* and patients were not re-contacted. All patient data analysed were anonymized.

## Results

### Cohort analysis

**Fig 1:**
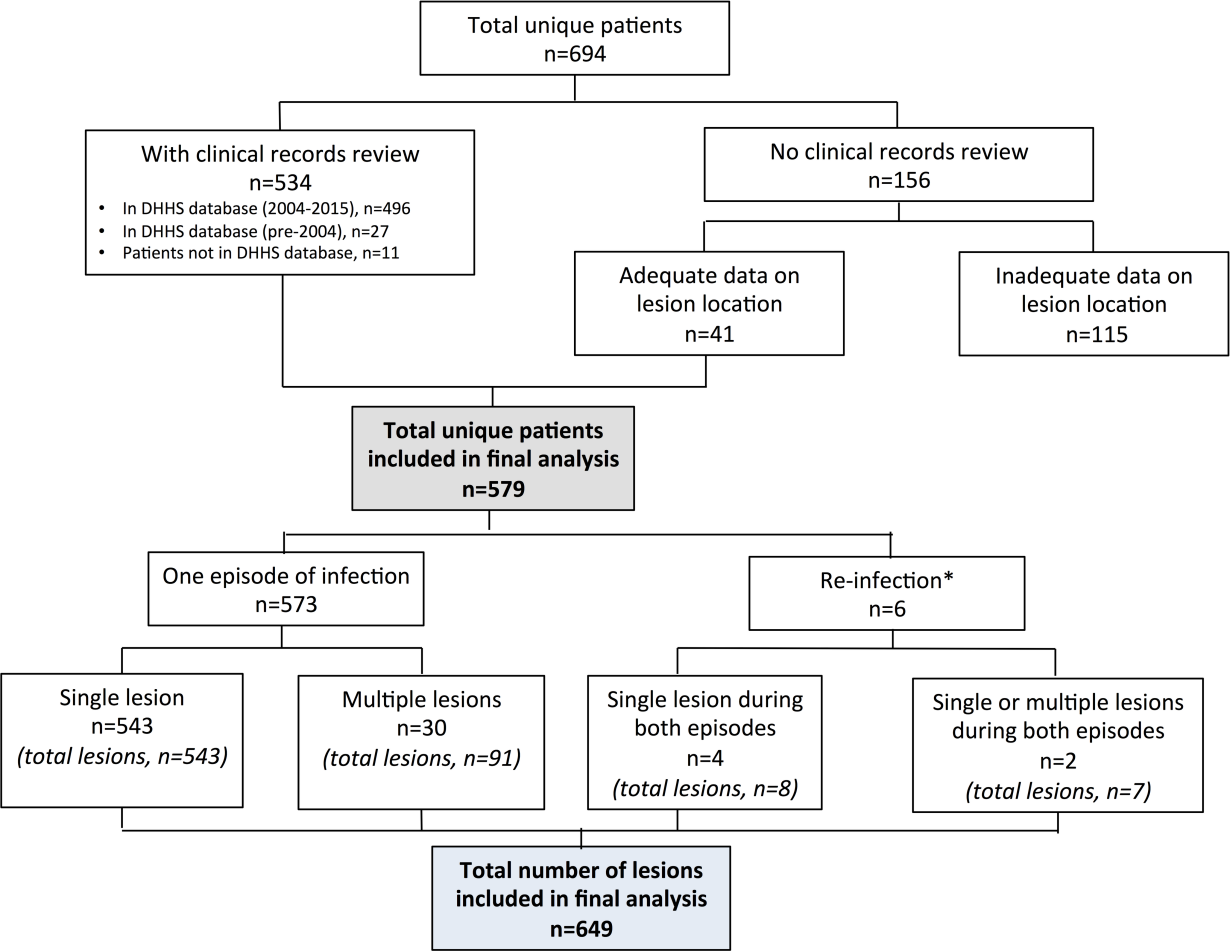
Flowchart of study participation. *Four patients had single lesions during both episodes. Two patients had a single lesion in one episode and multiple lesions in another episode. DHHS–Department of Health & Human Services.

Of the 694 patients diagnosed with BU during the study period, including 27 who were diagnosed prior to 2004, we have been able to review the clinical records of 538 patients (77·5%). Combined with an additional 41 patients with adequate data on lesion location, the final analysis included 579 patients (83·4%) who contributed to a total of 585 episodes of infection and 649 lesions (Fig 1). There were six patients (1·0%) with two episodes of infection that were more than 12 months apart and 32 patients (5·5%) with multiple lesions, including those with likely reinfection and multiple lesions (n = 2), with the median number of lesions being 2 (IQR 2–3; Range 2–13).

Among the 585 total episodes of infection, 54·5% (n = 319) were from male patients and 45·5% (n = 266) were from female patients. The median age was 55 years (interquartile range = 30 to 71 years) while the age range was 1 to 95 years. Children 15 years and under accounted for 12·8% of patients (n = 75) while 51·5% (n = 301) were between 15 and 65 years, and 35·7% (n = 209) greater than or equal to 65 years. Most patients (n = 538; 92·0%) reported exposure in one endemic region, with 67·5% (n = 363) exposed on the Bellarine Peninsula, 19·5% (n = 105) exposed on the Mornington Peninsula and 13·0% (n = 70) exposed in other regions. A further 6·5% (n = 40) reported exposure in more than one endemic location and no information about the location of exposure could be obtained for the remaining 7 (1.2%) patients.

### Lesion localisation

**Fig 2:**
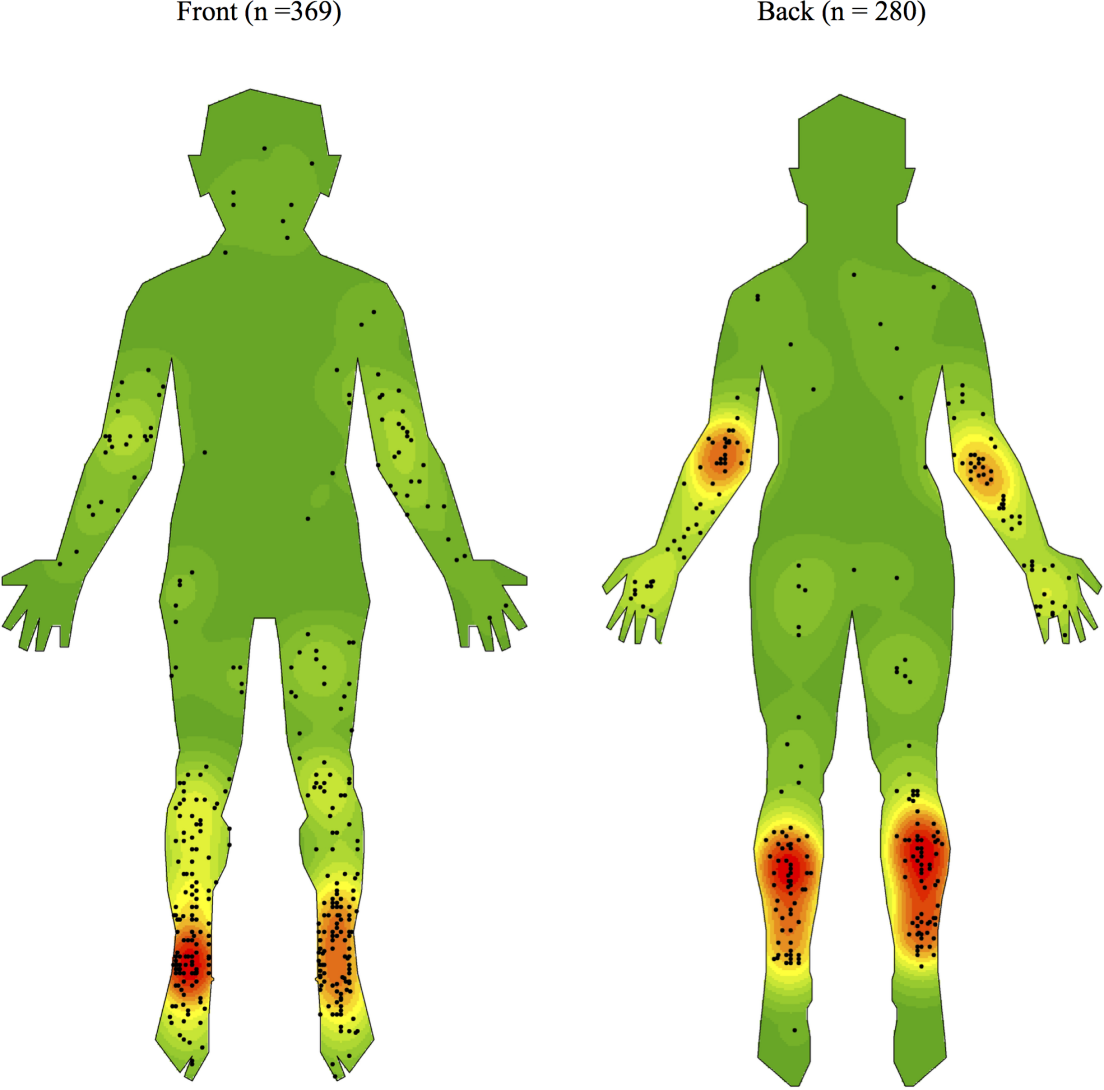
Density map of the distribution of Buruli ulcer lesions on front and back templates of the human body generated using ArcGIS software version 10.3.1.

**Fig 3:**
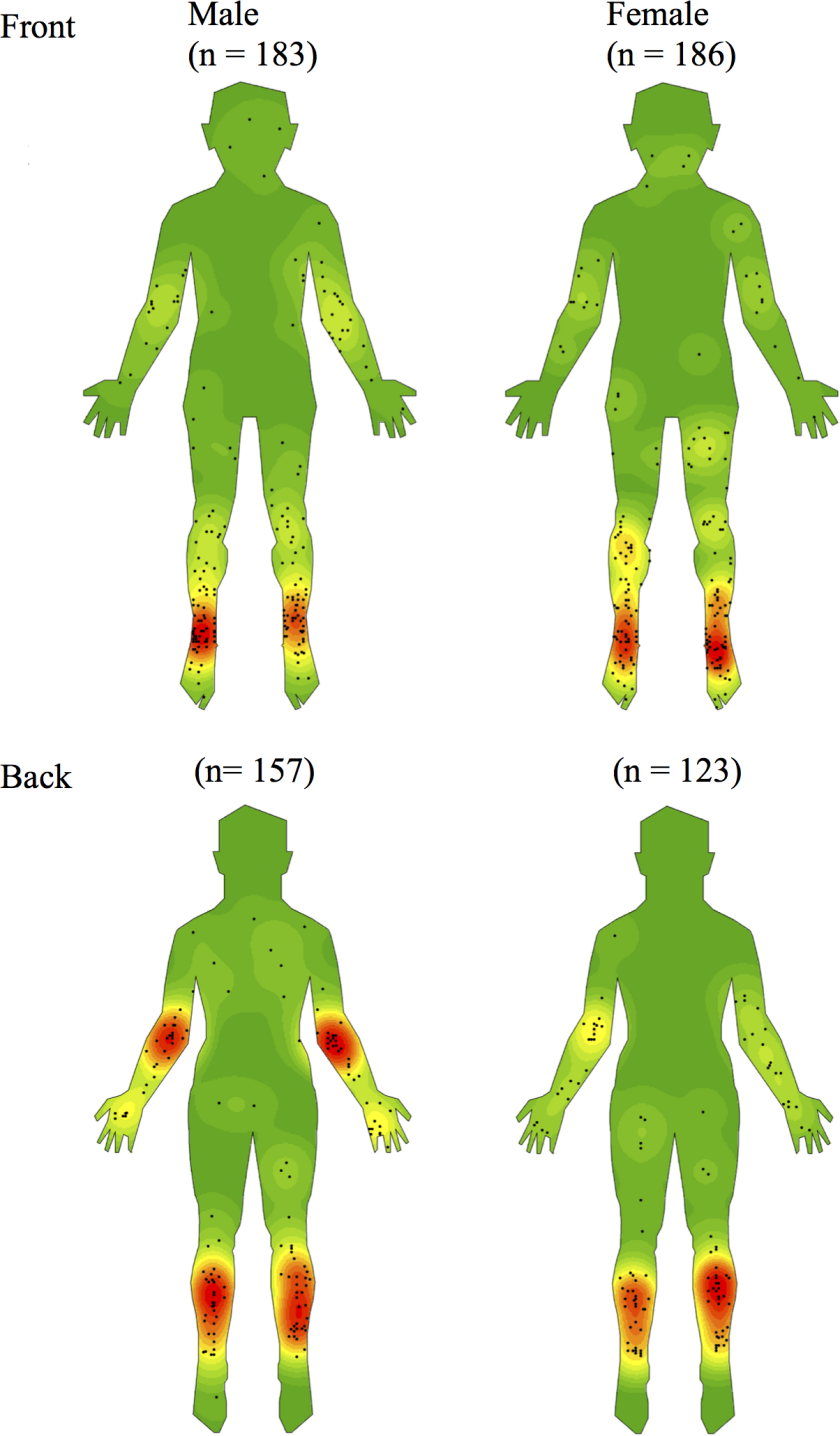
Density maps stratified by gender.

**Fig 4:**
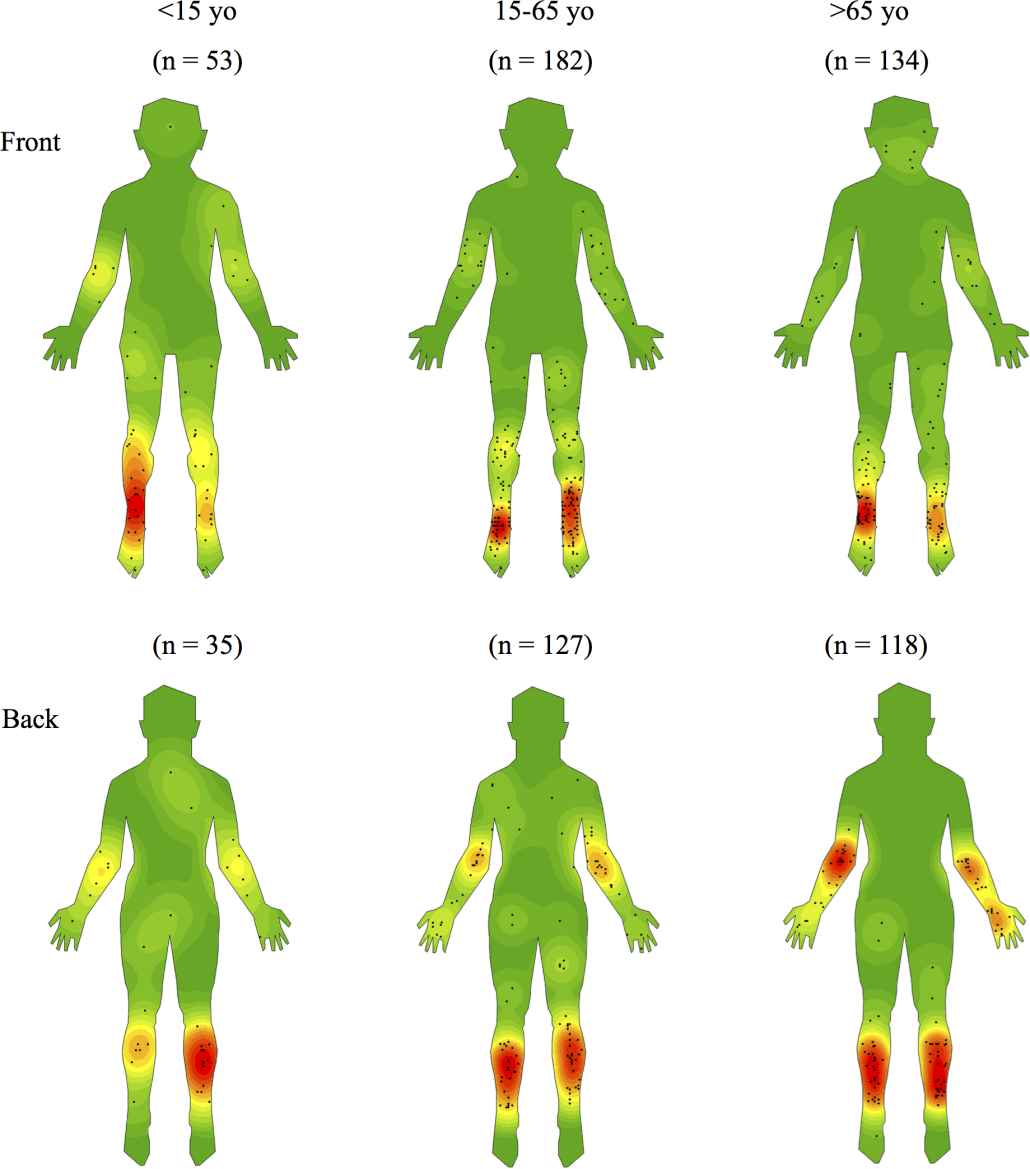
Density maps stratified by age.

**Fig 5:**
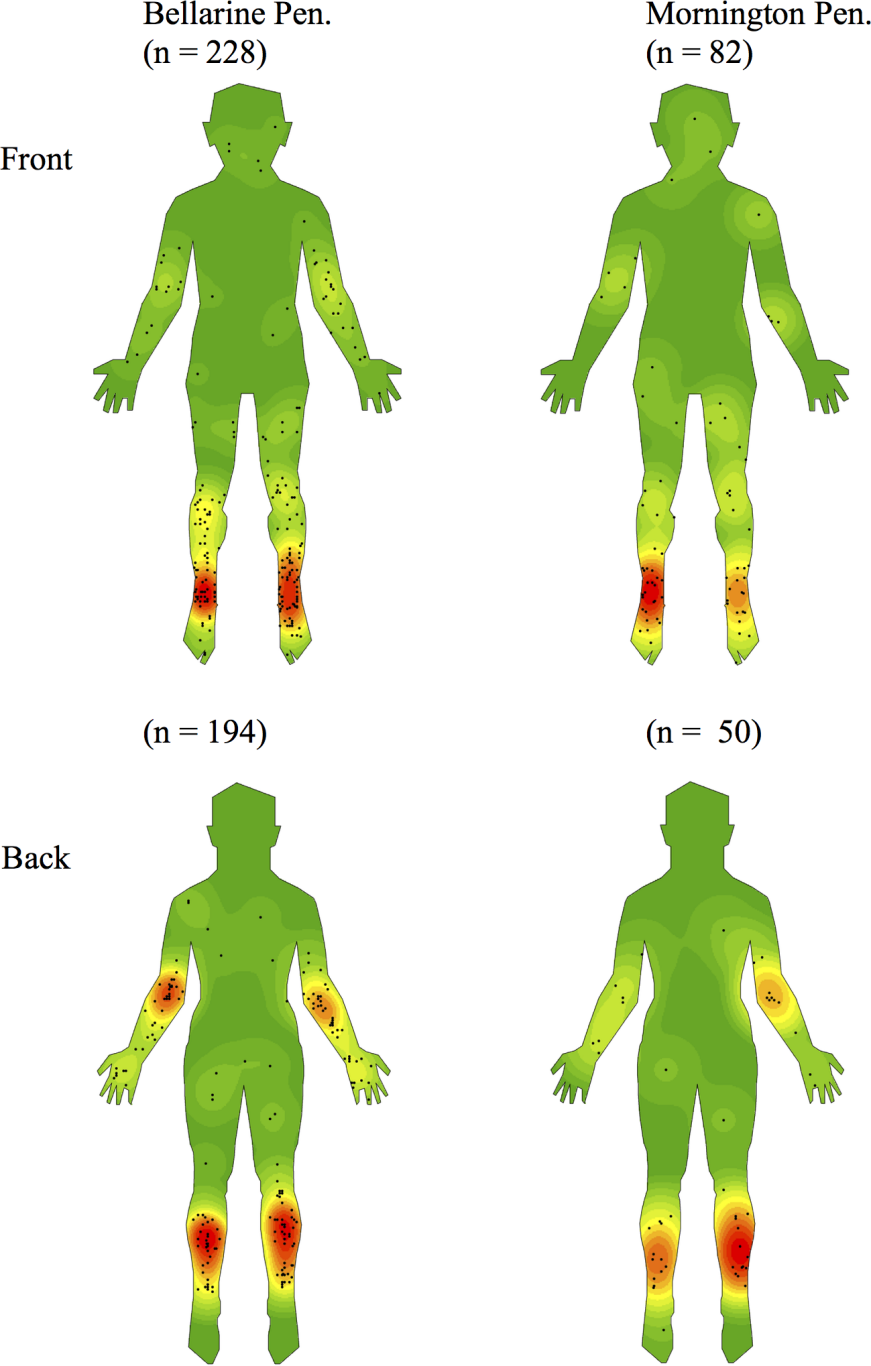
Density maps stratified by endemic region of exposure.

As shown in Figs 2–5 and Table 1, we observed a qualitatively non-random distribution of BU lesions on the human body. When comparing body regions (Fig 2 & Table 2), lesions were most common on the upper and lower limbs accounting for 27·1% and 70·0% of all lesions respectively. There were comparatively very few lesions on the trunk (1·7%) and the head or neck regions (1·2%). On limbs, it was the distal regions that were most affected. The calves and ankles showed the greatest density on the lower limb while the elbow and dorsal surfaces of the forearm and hand were the regions mainly affected on the upper limb.

**Table 1:** Number and percentages of BU lesions stratified by gender, age, exposure location and body regions.

	Gender		Age			Exposure location	
	Male	Females	≤15	15–65	≥65	BP	MP
	n (%)	n (%)	n (%)	n (%)	n (%)	n (%)	n (%)
**Body regions**	** **	** **	** **	** **	** **	** **	** **
Head and neck	4 (1.2)	4 (1.3)	1 (1.1)	1 (0.3)	6 (2.4)	5 (1.2)	3 (2.3)
Trunk	9 (2.6)	2 (0.6)	3 (3.4)	5 (1.6)	3 (1.2)	8 (1.9)	2 (1.5)
Upper limb	113 (33.2)	63 (20.4)	23 (26.1)	78 (25.2)	75 (29.8)	132 (31.3)	28 (21.2)
Lower limb	214 (62.9)	240 (77.7)	61 (69.3)	225 (72.8)	168 (66.7)	277 (65.6)	99 (75)
**Lower limbs**	**214**	**240**	**61**	**225**	**168**	**277**	**99**
Hip	1 (0.5)	1 (0.4)	1 (1.6)	0 (0)	1 (0.6)	1 (0.4)	1 (1)
Buttock	1 (0.5)	2 (0.8)	0 (0)	2 (0.9)	1 (0.6)	2 (0.7)	0 (0)
Thigh	17 (7.9)	23 (9.6)	6 (9.8)	22 (9.8)	12 (7.1)	22 (7.9)	12 (12.1)
Knee	20 (9.3)	25 (10.4)	9 (14.8)	29 (12.9)	7 (4.1)	28 (10.1)	8 (8.1)
Leg	113 (52.8)	103 (42.9)	32 (52.5)	100 (44.4)	84 (50)	132 (48.4)	48 (48.5)
Ankle	48 (22.4)	54 (22.5)	9 (14.8)	47 (20.9)	46 (27.4)	67 (24.2)	20 (20.2)
Foot	14 (6.5)	32 (13.3)	4 (6.6)	25 (11.1)	17 (10.1)	23 (8.3)	10 (10.1)
**Upper limbs**	**113**	**63**	**23**	**78**	**75**	**132**	**28**
Shoulder	2 (1.8)	3 (4.8)	1 (4.3)	4 (5.1)	0 (0)	3 (2.3)	1 (3.6)
Arm	17 (15)	16 (25.4)	5 (21.7)	22 (28.2)	6 (8)	23 (17.4)	8 (28.6)
Elbow	44 (38.9)	19 (30.2)	9 (39.1)	25 (32.1)	29 (38.7)	46 (34.8)	10 (35.7)
Forearm	26 (23)	10 (15.9)	6 (26.1)	14 (17.9)	16 (21.3)	30 (22.7)	5 (17.9)
Wrist	6 (5.3)	8 (12.7)	0 (0)	3 (3.8)	11 (14.7)	12 (9.1)	2 (7.1)
Hand	18 (15.9)	7 (11.1)	2 (8.7)	10 (12.8)	13 (17.3)	18 (13.6)	2 (7.1)
**Joints**							
Ankle	48 (14.1)	54 (17.5)	9 (10.2)	47 (15.2)	46 (18.3)	67 (15.9)	20 (15.2)
Elbow	44 (12.9)	19 (6.1)	9 (10.2)	25 (8.1)	29 (11.5)	46 (10.9)	10 (7.6)
Knee	20 (5.9)	25 (8.1)	9 (10.2)	29 (9.4)	7 (2.8)	28 (6.6)	8 (6.1)
Wrist	6 (1.8)	8 (2.6)	0 (0)	3 (1)	11 (4.4)	12 (2.8)	2 (1.5)
Shoulder	2 (0.7)	3 (1)	1 (1.1)	4 (1.3)	0 (0)	3 (0.7)	1 (0.8)
Hip	1 (0.3)	1 (0.3)	1 (1.1)	0 (0)	1 (0.4)	1 (0.2)	1 (0.8)

**Table 2:** Adjusted and unadjusted associations of *M*. *ulcerans* lesion body positions with age and gender.

Body site	Male gender: n (%)	Female gender: n (%)	OR male v female adjusted for age (95% CI)	p-value male v female adjusted for age	Age ≥ 65 years: n (%)	Age < 65 years: n (%)	OR age ≥ 65 years v age < 65 years adjusted for gender	p-value age ≥ 65 years v age < 65 years adjusted for gender
Upper limb	113 (33.2)	63 (20.4)	**1.97**	**<0.001**	75	101	1.29 (0.90-	0.16
			**(1.38-**		(29.8)	(25.4)	1.85)	
			**2.82)**					
Lower limb	214 (62.9)	240 (77.7)	**0.48**	**<0.001**	168	286	0.74 (0.52-	0.09
			**(0.34-**		(66.7)	(72.0)	1.05)	
			**0.68)**					
Proximal	289 (85.0)	258 (83.5)	1.15(0.75	0.52	6 (2.4)	32 (8.1)	**0.57 (0.36-**	**0.02**
			-1.76)				**0.91)**	
Arm and	19 (6.2)	19 (5.6)	0.86	0.65	6 (2.4)	32 (8.1)	**0.28 (0.11-**	**0.001**
shoulder			(0.44-				**0.67)**	
combined			1.66)					
Hand,	94 (27.7)	44 (14.2)	**2.41(1.61**	**<0.001**	69	69	**1.91 (1.30-**	**0.001**
wrist,			**-3.61)**		(27.4)	(17.4)	**2.81)**	
forearm								
and elbow								
combined								
Ankle	48 (14.1)	54 (17.5)	0.79	0.27	48	56	1.34 (0.88-	0.18
			(0.51-		(18.3)	(14.1)	2.06)	
			1.20)					
Thigh	17 (5.0)	23 (7.4)	0.64	0.18	12 (4.8)	28 (7.1)	0.64 (0.32-	0.22
			(0.33-				1.29)	
			1.22)					
Foot	14 (4.1)	32 (10.4)	**0.37**	**0.002**	17 (6.8)	29 (7.3)	0.87 (0.47-	0.67
			**(0.19-**				1.63)	
			**0.71)**					
Joint	120 (35.3)	109 (35.3)	1.00	0.97	93	136	1.12 (0.81-	0.49
			(0.73-		(36.9)	(34.3)	1.56)	
			1.39)					

**Fig 6:**
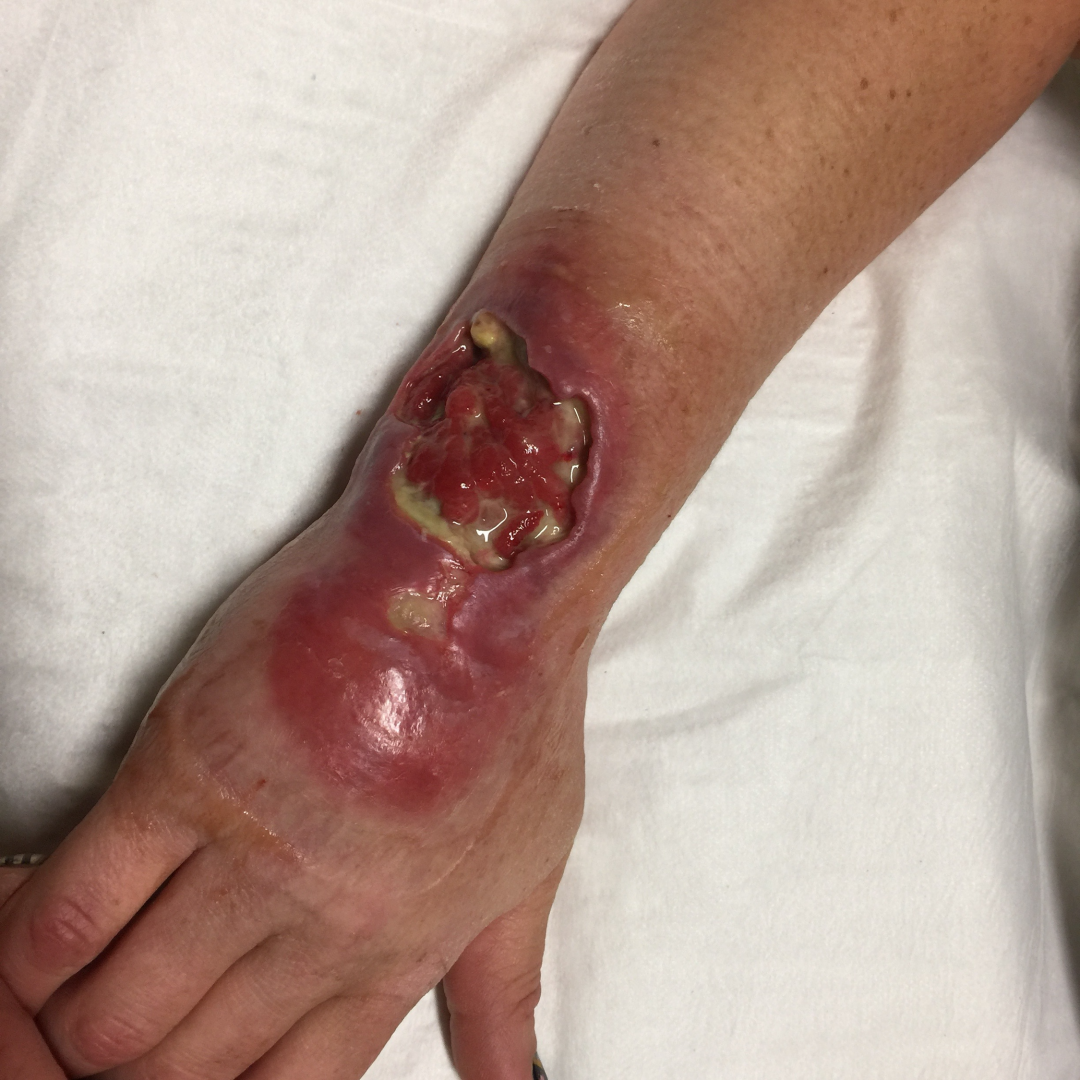
Right forearm of young woman with category III Buruli ulcer acquired on the Mornington Peninsula.

There were no obvious differences comparing the right or left side of the body with similar density patterns on each. There were two midline lesions from two different children, one at the natal cleft and the other on the nose. Notably, only one lesion was found on the sole of the foot and none were found on the palms of the hands. Lesions were over a joint in 35.6% of cases. Comparing distribution between large joints of the upper and lower limbs (Table 1), the ankles accounted for 15·7% (n = 102) while the elbow and knees were the next most affected with 9·7% (n = 63) and 6·9% (n = 45) of all lesions respectively. An example of a severe case of Buruli ulcer crossing a joint and acquired on the Mornington Peninsula is shown in Fig 6.

### Comparisons by gender, age and endemic exposure region

When stratified by gender and adjusted for age, the odds of males having a lesion on the upper limb was almost twice that compared to females (OR 1·97, 95% CI 1·38–2·82, p<0·001) but halved when comparing with females on the lower limbs (OR 0.48, 95% CI 0.34–0.68, p<0.001). Males also had a significantly higher likelihood of having lesions on distal regions of the upper limb, that is, the hand, wrist, forearm and elbow combined (OR 2·41, 95% CI 1·61–3·61, p<0·001) but a significantly lower likelihood of having lesions on the foot (OR 0·37, 95% CI 0·19–0·71, p = 0·002).

When stratified by age and adjusted for gender, patients aged ≥65 years compared to those <65 years were significantly less likely to have proximal lesions (OR = 0·57, 95% CI 0·36–0.91, p = 0·02), significantly less likely to have a lesion on the arm and shoulder combined (OR = 0·28, 95% CI 0·11–0·67, p = 0·001) but significantly more likely to have a lesion on the hand, wrist, forearm and elbow combined (OR 1·91, 95% CI 1·30–2·81, p = 0·001).

Qualitative comparisons in BU distribution revealed no obvious differences between exposure on the Bellarine and Mornington Peninsulas (Fig 5).

### Multiple lesions

Of the 32 patients who had multiple lesions at the time of presentation, 17 (53%) had lesions located on separate limbs. Patients aged ≥ 65 years (OR 3·22, 95% CI 1·53–6·78, p = 0·001) and those with a lesion on the ankle (OR 2·71, 95% CI 1·23–5·98, p<0.01) were significantly more likely to have multiple lesions. There was no significant difference when comparing by gender in this regard (male compared with female OR 1.08, 95% CI 0.52–2.21, p = 0.84).

### Temperature and likely date of infection

The probable date of infection was estimated for 338 (59%) patients. These patients were grouped in 3-month intervals from hottest to coldest in descending order as shown in Table 3. Lesions on the arm and shoulder combined (OR 3·37, 95% CI 1·45–7·86, p = 0·003), as well as proximal lesions (OR 1·78, 95% CI 0·98–3·21, p = 0·05), were significantly associated with the hottest 3 months (Dec-Feb: 285 cases) when compared to the coldest 6 months (May-Oct: 103 cases).

**Table 3:** Likely calendar month of *M*. *ulcerans* infection categorised by maximal monthly temperatures.

Months	Average maximal daily temperature range (ºC)	Number of patients
December, January, February	24.2–26.0	132 (39.1%)
March, April, November	20.3–23.9	109 (32.2%)
May, September, October	16.7–19.7	56 (16.6%)
June, July, August	13.5–15.0	41 (12.1%)

## Discussion

We have shown a focal, non-random distribution of BU on the human body in Victoria, a Buruli endemic region with increasing disease incidence and a temperate climate. Areas of the body most affected include the ankles, calves, elbows, knees, and dorsal surfaces of the hands and forearms. BU was rare on the palms of the hands, soles of the feet, head, neck, and trunk. Combined, these findings suggest BU lesions are generally found on the distal regions of the upper and lower limbs, except the palms and soles, particularly around large joints. Our findings are similar to previous research on lesion localisation and the similarity in BU distribution in Victoria when compared with studies carried out in Africa raises the possibility of a unifying mechanism of transmission worldwide [6,11].

The distribution pattern of BU we have observed appears to correlate with exposed skin areas not covered by clothing. Indeed, a failure to wear protective clothing has been previously documented as a risk factor for developing the disease [15]. The warmer summer months of the year may therefore be periods of high exposure risk when less clothing is worn. This is supported by our findings of an increased proportion of cases likely acquired in warmer months, and the fact that warmer months were associated with an increased likelihood of having lesions on proximal body regions that are less likely to be protected by clothing during these periods.

Stratification by age and gender revealed further differences in distribution. Compared to younger individuals, those aged greater than 65 had fewer lesions on proximal areas of the upper limbs. Similar findings have been noted in Africa where children and young adults were found to have more proximal lesions than those older [12]. Again, this may be due to clothing choices as older individuals may tend to wear longer sleeved upper garments. Comparing between genders revealed that upper limb lesions were more common in males, particularly at the elbows, while lower limb lesions were more common in females, especially the dorsum of the foot. This may be due to females choosing to wear more open footwear and longer-sleeved upper garments. Alternatively, males may be more prone to trauma, for example, when working as manual labourers. Future research is required to further explore these hypotheses.

While the exact mechanism of BU transmission remains unclear, an inoculating event such as direct trauma or insect bites is commonly thought of as a pre-requisite for disease emergence [15]. One theory of transmission is inoculation through direct contact with contaminated environmental sources such as sharp leaves or thorns or through existing wounds [4]. It is plausible that random trauma through environmental contact could produce a specific distribution pattern of BU. Additionally, previous research has shown body regions such as the ankles, shins and elbows, where skin lies close to bone, as being more prone to injury in children [16]. However, we have also observed BU to cluster at the calves, regardless of age. The predilection for BU on this relatively protected part of the body may therefore suggest transmission in this body region is less likely to occur via existing wounds or casual trauma.

We feel our data from Victoria fits best with the hypothesis that *M*. *ulcerans* is transmitted via insect vectors such as mosquitoes [4,17,18]. With the recent discovery that possums, a mammal native to Australia, can harbour *M*. *ulcerans* and subsequently develop clinical disease, it has been proposed that mosquitoes act as mechanical vectors connecting possums with humans living nearby [10,17]. Mosquitoes use a variety of visual, chemical and thermal cues to target a suitable location for blood meals in humans and published data exists showing a species-dependent selection of biting sites which are characteristically non-random [19,20]. As BU occurs mainly at exposed large joints and the back of the legs, these areas could be targets for Australian mosquito populations in endemic zones. Mapping of species-specific mosquito biting preferences and comparison with our density maps could provide a unique opportunity to test this hypothesis. Future research could also compare our maps with whole body thermographs to examine the theory that *M*. *ulcerans* has a preference for cooler body sites.

We accept that there may be different modes of Buruli transmission in different regions of the world. Studies of lesion location performed in Africa have sometimes shown a predominance of right-sided lesions, suggesting that acquisition of infection follows contact between the preferred leading arm or leg and a contaminated environment [21,22]. However we did not find evidence for this in our study nor is this pattern uniform in all African case series [23]. So far, investigation of field captured mosquitoes [24] and small animals [25] in Africa has not identified an analogue to the possum-amplifier-mosquito-vector scenario we have proposed for southern Australia [17]. Nevertheless, the generally held view that *M*. *ulcerans* is widespread and free-living in endemic environments and that direct contact is all that is required to acquire infection needs to be challenged. This view is not supported by our findings, or those from Africa showing sparing of the soles of the feet and palms of the hand. Furthermore, analysis of the whole genome sequence of *M*. *ulcerans* suggests a niche-adapted rather than free-living existence, probably in association with other biota, as there are more than 700 gene deletions or interruptions compared with its *M*. *marinum* progenitor [26]. In support of this prediction *M*. *ulcerans* has only been isolated in pure culture directly from the environment once, and that was from a water insect captured in Benin [27]. Furthermore, while there are differences, there are also similarities between our results and the distribution of lesions reported by Bratschi *et al* in Cameroon on which we have based this research [11]. Hence it is possible that biting insects are the predominant mode of transmission of *M*. *ulcerans* everywhere. If true, prevention of insect bites should substantially reduce the incidence of Buruli ulcer in people living in endemic areas.

Limitations of our research include being unable to collect data on approximately 17% of the total number of patients notified as cases during our study period. However, we have mapped a large cohort of patients and it is unlikely missing cases would significantly alter our principal findings. There may also be a possible loss of spatial resolution due to the 2-dimensional nature of our heat-map representation. In total, there were 185 lesions (28·5%) which that were located on the medial or lateral sides of the body. Nevertheless, our interest in lesion localisation was the anterior or posterior aspects of a body region or joint and our mapping process was standardised to include side lesions on the front template only, for easier visualisation.

In conclusion, our results provide clear evidence for a highly specific, non-random distribution of BU lesions on the human body. Our study was significantly larger than a BU lesion distribution study performed in Cameroon but we found a generally similar pattern suggesting *M*. *ulcerans* transmission and pathogenesis may be similar across the world despite very different geographical and climatic conditions. Our study will also inform clinicians who need to consider the differential diagnosis of skin lesions in routine clinical practice by being able to compare with our density maps, as well as guide future researchers interested in understanding disease transmission and its prevention.

## Supporting information

S1 Checklist STROBE checklist. (PDF)
